# Primary Pericardial Synovial Sarcoma Presenting as a Giant Intrapericardial Mass: A Case Report

**DOI:** 10.1155/cric/2308191

**Published:** 2026-11-15

**Authors:** Xiang Zhou, Hongyan Liu, Wenqiang Sun, Zhengdong Hua

**Affiliations:** ^1^ Department of Cardiac Surgery Wuhan Asia Heart Hospital Wuhan China wahh.com.cn; ^2^ Wuhan Clinical Center for Cardiomyopathy Wuhan China; ^3^ Hubei Province Key Laboratory of Occupational Hazard Identification and Control Wuhan University of Science and Technology Wuhan China wust.edu.cn

**Keywords:** fluorescence in situ hybridization, intrapericardial mass, multimodality imaging, primary pericardial tumor, SS18 rearrangement, synovial sarcoma

## Abstract

Primary pericardial synovial sarcoma is an extremely rare malignant tumor with nonspecific presentation and poor prognosis. We report the case of a 29‐year‐old woman whose pericardial mass was detected during a workplace health examination. Transthoracic echocardiography revealed a giant intrapericardial mass predominantly compressing the left atrium. Computed tomography and magnetic resonance imaging demonstrated a heterogeneous intrapericardial mass extending from the left margin of the main pulmonary artery to the left atrioventricular groove, with partial invasion of the left atrial cavity. Ultrasound‐guided biopsy showed a monophasic spindle‐cell neoplasm. Immunohistochemistry showed focal weak positivity for pancytokeratin and epithelial membrane antigen, positivity for vimentin and D2‐40, weak positivity for CD99, and a Ki‐67 labeling index of approximately 15%. Fluorescence in situ hybridization using an SS18 break‐apart probe demonstrated split red and green signals in tumor‐cell nuclei, consistent with SS18 rearrangement. The patient declined surgical resection and adjuvant therapy and died 22 months after diagnosis. This case emphasizes that multimodality imaging is essential for defining tumor extent but cannot establish histological subtype. Integrated histology, immunohistochemistry, and SS18 fluorescence in situ hybridization are required for diagnostic confirmation.

**Learning Points**

• Primary pericardial synovial sarcoma may be detected incidentally during a workplace health examination.
• Multimodality imaging is essential for defining the anatomical extent of a giant intrapericardial mass, but imaging cannot establish the histological subtype.
• Integrated histology, immunohistochemistry, and SS18 break‐apart fluorescence in situ hybridization are required to support the diagnosis of monophasic synovial sarcoma.

## 1. Introduction

Primary tumors of the pericardium are rare, and malignant entities represent a small proportion of these cases [1]. Synovial sarcoma is an aggressive soft tissue malignancy that typically arises in the extremities and only rarely involves the heart or pericardium [2]. Primary pericardial synovial sarcoma is therefore difficult to recognize because its symptoms are nonspecific, its anatomical relationships are complex, and its imaging appearance overlaps with other pericardial tumors [3–5].

We present a case of a giant primary pericardial synovial sarcoma that was detected during a workplace health examination and produced marked compression of the left atrium. The novelty and learning value of this case lie in the combination of an unusual clinical presentation, detailed multimodality imaging, and diagnostic confirmation through an integrated pathological workup, including histology, immunohistochemistry, and SS18 fluorescence in situ hybridization [2, 3, 6].

## 2. Case Presentation

A 29‐year‐old woman with no significant past medical history had fever in October 2019, without chest pain, chest tightness, or dyspnea. Echocardiography at another hospital showed a pericardial effusion. The fever resolved after approximately 5 days of treatment, and repeat echocardiography in November showed a marked reduction in the effusion. On December 3, a workplace health examination identified a pericardial mass on echocardiography, prompting referral to our hospital. She was admitted on December 13 with a temperature of 36.5°C and no signs of heart failure.

Transthoracic echocardiography showed a giant mass within the pericardial space, predominantly located over the left atrium (Figure 1). Contrast‐enhanced computed tomography revealed a mixed‐density mass measuring 101 × 76 × 96 mm, extending from the left margin of the main pulmonary artery to the left atrioventricular groove, with compression of the left atrium and left lower pulmonary vein (Figure 2). Magnetic resonance imaging demonstrated a heterogeneous intrapericardial mass surrounding the ascending aorta and main pulmonary artery, with partial invasion of the left atrial cavity (Figure 3).

**Figure 1:**
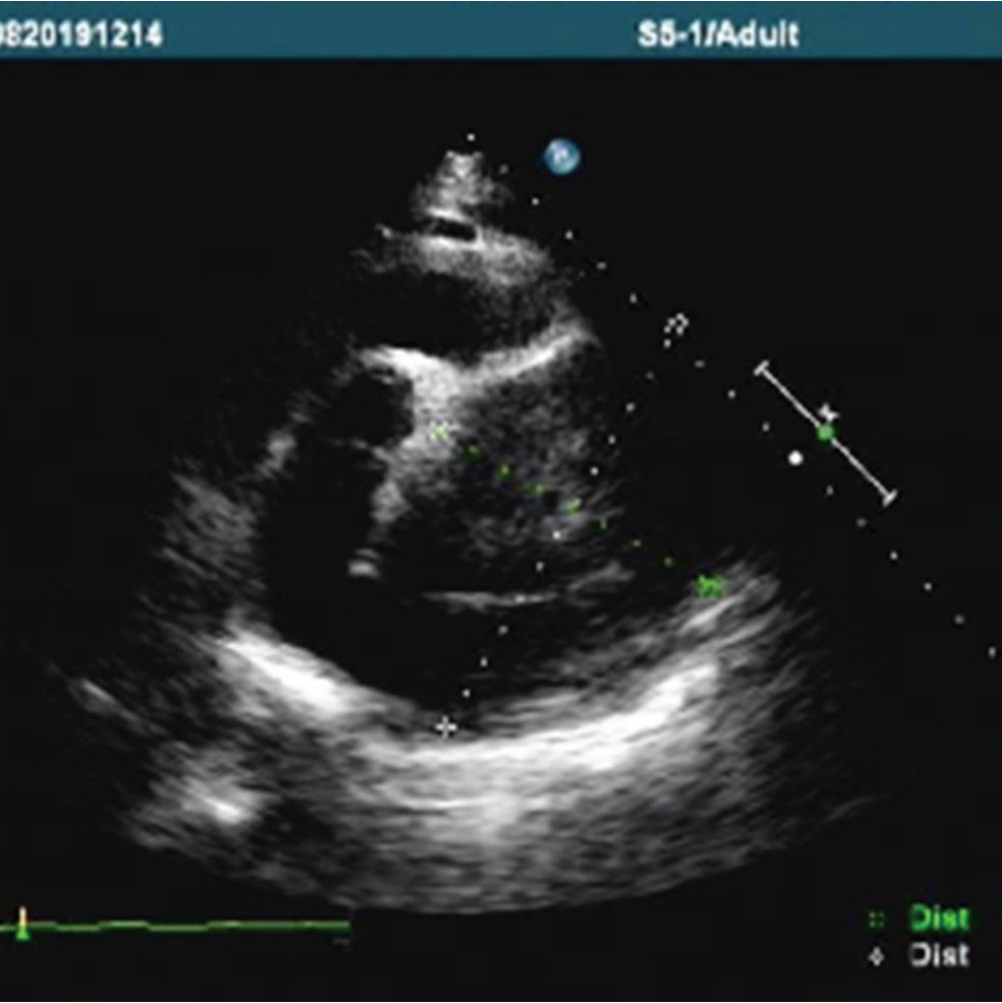
Transthoracic echocardiography demonstrating a large intrapericardial mass predominantly compressing the left atrium.

**Figure 2:**
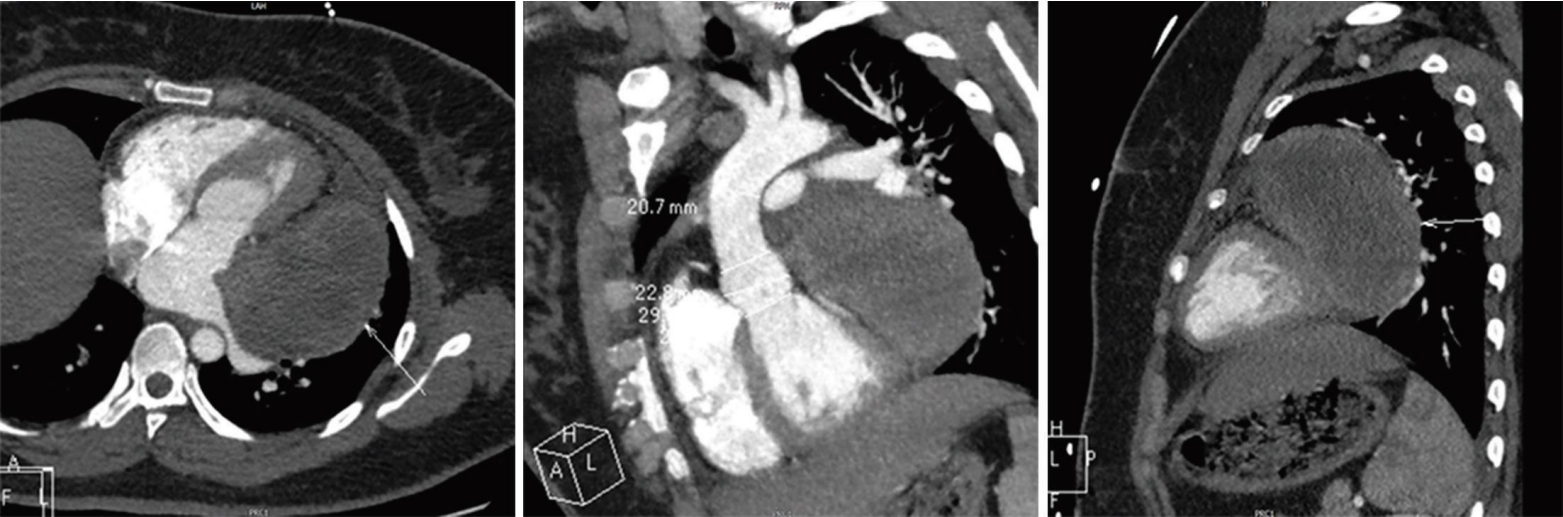
Contrast‐enhanced computed tomography showing a heterogeneous intrapericardial mass extending from the left margin of the main pulmonary artery to the left atrioventricular groove, with compression of the left atrium and left lower pulmonary vein.

**Figure 3:**
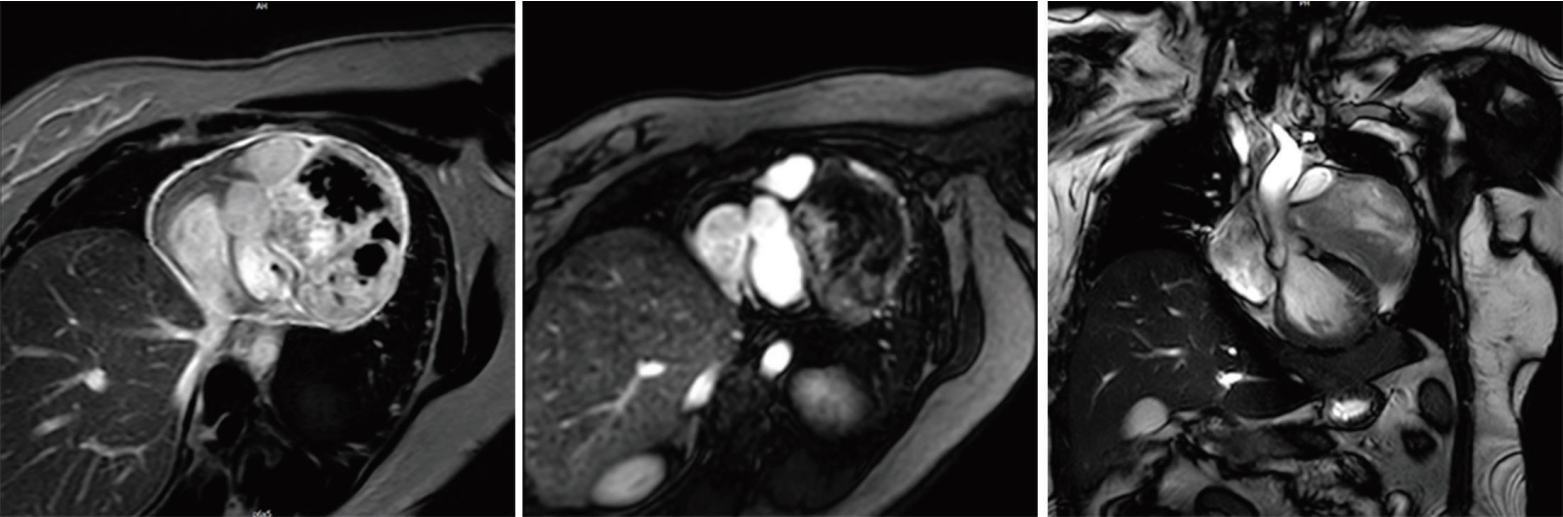
Cardiac magnetic resonance imaging revealing a heterogeneous intrapericardial mass surrounding the ascending aorta and main pulmonary artery, with partial invasion of the left atrial cavity.

An ultrasound‐guided pericardial biopsy was performed. Histopathological examination showed a densely cellular spindle‐cell neoplasm with a monophasic pattern. The tumor cells were arranged in fascicles and short bundles on hematoxylin and eosin staining (Figure 4A).

**Figure 4:**
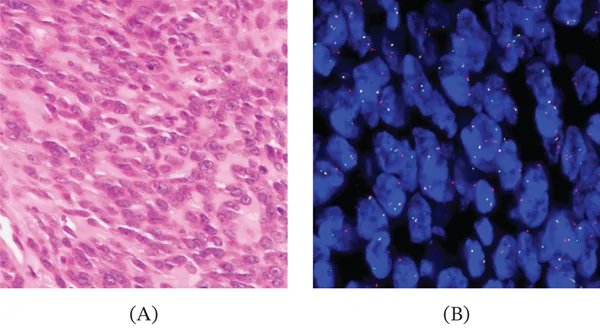
Histopathological and molecular findings. (A) Hematoxylin and eosin staining showing a monophasic spindle‐cell neoplasm composed of spindle cells arranged in fascicles and short bundles. (B) SS18 break‐apart fluorescence in situ hybridization showing split red and green signals in tumor‐cell nuclei, consistent with SS18 gene rearrangement.

Immunohistochemically, the tumor cells showed focal weak positivity for pancytokeratin and epithelial membrane antigen, positivity for vimentin and D2‐40, weak positivity for CD99, and focal positivity for h‐caldesmon. The tumor cells were negative for smooth muscle actin, desmin, CD34, CD56, MyoD1, CK5/6, calretinin, WT‐1, PLAP, CD3, CD20, CD45, and myeloperoxidase. The Ki‐67 labeling index was approximately 15%.

Fluorescence in situ hybridization using an SS18 break‐apart probe demonstrated split red and green signals in tumor‐cell nuclei, consistent with SS18 gene rearrangement (Figure 4B). Taken together, the monophasic spindle‐cell morphology, immunohistochemical profile, and SS18 rearrangement supported the diagnosis of monophasic synovial sarcoma [2, 3, 6].

Surgical resection and adjuvant therapy were recommended; however, the patient declined further treatment. She died 22 months after the initial diagnosis.

## 3. Discussion

This case provides several practical learning points for clinicians evaluating pericardial masses. First, primary pericardial synovial sarcoma may be detected incidentally during a workplace health examination. Second, a large intrapericardial tumor can extend along complex cardiovascular structures and partially invade the cardiac chamber. Third, radiological findings are insufficient to establish the histological subtype, making tissue diagnosis and molecular confirmation essential.

Multimodality imaging played an essential role in evaluating the extent of the lesion and its relationship with adjacent cardiac structures. Transthoracic echocardiography allowed initial detection of the mass, whereas computed tomography and magnetic resonance imaging provided further anatomical and tissue characterization. However, imaging findings alone were insufficient to establish a definitive diagnosis, as the appearance overlapped with other pericardial tumors [3, 4].

The pathological workup was central to the diagnosis. The monophasic spindle‐cell morphology and focal epithelial marker expression were compatible with synovial sarcoma, but the differential diagnosis included other spindle‐cell tumors involving the pericardium. Although D2‐40 was positive, this result was interpreted in the context of the full immunohistochemical and molecular profile rather than as stand‐alone evidence of mesothelial differentiation. Negative staining for calretinin and WT‐1, together with focal epithelial marker expression and SS18 rearrangement, supported synovial sarcoma rather than malignant mesothelioma. The absence of CD34, desmin, and hematolymphoid markers also helped exclude other important mimics. The demonstration of SS18 rearrangement by break‐apart fluorescence in situ hybridization provided molecular support for the final diagnosis [2, 3, 6]. This integrated approach is especially important in rare cardiac and pericardial tumors, where morphology and imaging alone may be insufficient.

Complete surgical resection is generally considered the main therapeutic option for synovial sarcoma when anatomically feasible [4, 5]. In this patient, surgery and adjuvant therapy were recommended, but treatment was declined. The disease subsequently followed a progressive course, and the patient died 22 months after diagnosis. This outcome underscores the aggressive behavior of primary pericardial synovial sarcoma and the importance of early diagnostic confirmation, multidisciplinary treatment planning, and careful discussion of therapeutic options [4, 5].

## 4. Conclusion

This case illustrates the diagnostic challenges and clinical learning points associated with primary pericardial synovial sarcoma. Multimodality imaging is valuable for defining the anatomical extent of disease. However, diagnostic confirmation requires an integrated pathological assessment, including histology, immunohistochemistry, and SS18 fluorescence in situ hybridization. Awareness of this rare entity may help clinicians include synovial sarcoma in the differential diagnosis of large intrapericardial masses with nonspecific symptoms.

## Author Contributions

Xiang Zhou, Hongyan Liu, and Wenqiang Sun contributed equally to this work.

## Funding

This study was supported by the National Science and Technology Major Project for Noncommunicable Chronic Diseases (2023ZD0504503); the Natural Science Foundation of Wuhan (2024040801020377); and the Joint Research Fund of Hubei Province Key Laboratory of Occupational Hazard Identification and Control (JF2023‐K03).

## Disclosure

All authors take full responsibility for the manuscript.

## Ethics Statement

Ethical approval was not required for this single‐patient case report according to institutional policy.

## Consent

Written informed consent was obtained for publication of this case report and accompanying images.

## Conflicts of Interest

The authors declare no conflicts of interest.

## Data Availability

Data sharing is not applicable to this article as no datasets were generated or analyzed during the current study.
